# *Cymbomonas tetramitiformis -* a peculiar prasinophyte with a taste for bacteria sheds light on plastid evolution

**DOI:** 10.1007/s13199-016-0464-1

**Published:** 2016-11-10

**Authors:** Przemysław Gagat, Paweł Mackiewicz

**Affiliations:** Department of Genomics, Faculty of Biotechnology, University of Wrocław, ul. Joliot-Curie 14A, 50-383 Wrocław, Poland

**Keywords:** *Cymbomonas*, Endosymbiosis, Green algae, Mixotrophy, *Paulinella*, Phagocytosis

## Abstract

*Cymbomonas tetramitiformis* is a peculiar green alga that unites in one cell the abilities of photosynthesis and phagocytosis, which makes it a very useful model for the study of the evolution of plastid endosymbiosis. We have pondered over this issue and propose an evolutionary scenario of trophic strategies in eukaryotes, including primary and secondary plastid endosymbioses. *C. tetramitiformis* is a prototroph, just like the common ancestor of Archaeplastida was, and can synthesize most small organic molecules contrary to other eukaryotic phagotrophs, e.g. some metazoans, amoebozoans, and ciliates, which have not evolved tight endosymbiotic relationships. In order to establish a permanent photosynthetic endosymbiont they do not have to become prototrophs, but have to acquire the genes necessary for plastid retention via horizontal (including endosymbiotic) gene transfer. Such processes occurred successfully in the ancestors of eukaryotes with permanent secondary plastids and thus led to their great diversification. The preservation of phagocytosis in *Cymbomonas* (and some other prasinophytes as well) seems to result from nutrient deficiency in their oligotrophic habitats. This forces them to supplement their diet with phagocytized prey, in contrasts to the thecate amoeba *Paulinella chromatophora*, which also successfully transformed cyanobacteria into permanent organelles. Although *Paulinella* endosymbionts were acquired very recently in comparison to primary plastids, *Paulinella* has lost the ability to phagocytose, most probably due to the fact that it inhabits nutrient-rich environments, which renders the phagotrophy nonessential.

## Introduction

About 1.5 billion years ago, a phagotrophic eukaryote acquired a cyanobacterium that was subsequently transformed into the two-membrane-bounded photosynthetic organelle called a primary plastid (Parfrey et al. 2011; McFadden 2014; Zimorski et al. 2014). Before the transformation, however, the cyanobacteria were hunted and phagocytized by their prospective eukaryotic host. This initially hostile interaction triggered the evolution of Archaeplastida (Plantae), a supergroup of eukaryotes that comprises three photosynthetic lineages: glaucophytes (Glaucophyta), red algae (Rhodophyta), and green algae & higher plants (Viridiplantae) (De Clerck et al. 2012; Löffelhardt 2014; Mackiewicz and Gagat 2014).

Recently, a draft genome sequence of *Cymbomonas tetramitiformis,* a marine green alga with a peculiar trophic strategy, has been published (Burns et al. 2015). This early diverging prasinophyte not only performs photosynthesis, as the other archaeplastidians do, but also feeds by phagocytosis like a typical heterotroph and the presumed Archaeplastida progenitor (Moestrup et al. 2003; Maruyama and Kim 2013). Interestingly, bacterivorous prasinophytes have also been found in the genera of *Pterosperma*, *Halosphaera, Pyramimonas,* and *Micromonas* (Inouye et al. 1990; O’Kelly 1992; Bell and Laybourn-Parry 2003; McKie-Krisberg and Sanders 2014). Due to their mixotrophy, these green algae may represent an ancestral state of Archaeplastida evolution and thus help to understand the process of endosymbiosis and plastid evolution in general.

The ancestral nature of *C. tetramitiformis* is not only supported by its phylogenetic position among prasinophytes and by its feeding strategy, but also by the fact that its genome carries genes involved in lipid-A and peptidoglycan metabolism, two components of the bacterial cell wall derived from the cyanobacterial endosymbiont (Moestrup et al. 2003; Leliaert et al. 2012; Maruyama and Kim 2013; Burns et al. 2015). These genes, although uncommon, are also present in some other representatives of green plants (e.g. the green alga *Micromonas pusilla*, the moss *Physcomitrella patens*), and their phylogenies indicate that they are evolutionarily conserved among Viridiplantae species and have originated from a common ancestor (Burns et al. 2015). Genes involved in peptidoglycan (but not lipid-A synthesis) were also found in Glaucophyta, however, their plastids have additionally retained a clear peptidoglycan layer between the outer and inner plastid membranes (Pfanzagl et al. 1996; Jackson et al. 2015). Interestingly, Hirano et al. (2016) have recently visualized a peripherally distributed peptidoglycan layer in *P. patens* plastids using confocal microscopy, and Sato and colleagues (University of Tokyo, personal communication) have pinpointed its location to the intermembrane space applying a new method involving electron microscopy and density analysis of digital images at the resolution of 1 pixel. Their results indicate that the peptidoglycan layer might still be present in other Viridiplantae species, including *C. tetramitiformis*, and that it was lost independently at least three times in the Archaeplastida evolution: once in red algae, and twice in the green lineage, i.e. in chlorophytes and land plants (Takano and Takechi 2010).

The presence of lipid-A and peptidoglycan metabolism among members of green plants, and the mixed mechanism of feeding found in bacteriovorous prasinophytes (a *sine qua non* for plastid acquisition) suggest that the Viridiplantae clade might be the earliest branch of the Archaeplastida as some phylogenies indicate (e.g. the trees based on rRNA operon genes), although this contrasts with the prevailing belief that the root of the supergroup lies within Glaucophyta (see Mackiewicz and Gagat 2014 and references therein, and Jackson et al. 2015). The latter hypothesis is based on trees of nuclear genes and is supported by the presence of many ancestral traits typical of cyanobacteria preserved in glaucophyte plastids, including the peptidoglycan layer and unstacked thylakoid membranes with phycobilisomes (Mackiewicz and Gagat 2014; Jackson et al. 2015). Since these and other ancient characteristics represent preserved rather than derived traits, they do not lend decisive support for the Viridiplantae-first or Glaucophyta-first evolutionary scenarios. This issue could be resolved by phylogenetic analyses but their results are still controversial (see Mackiewicz and Gagat 2014 and references therein). More sequence data is required from glaucophytes and other poorly studied eukaryotic lineages, as well as new computational methods to eliminate stochastic and systematic errors in phylogenies (Mackiewicz and Gagat 2014). Nevertheless, the *Cymbomonas* case can provide interesting insight into the acquisition of permanent plastids and endosymbiosis in general.

## Gene transfer and plastid establishment

**Fig. 1:**
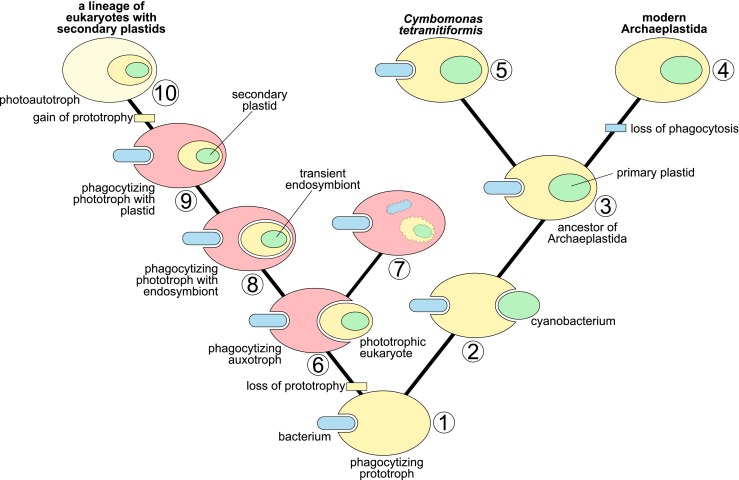
Evolution of trophic strategies in eukaryotes in the context of plastid endosymbiosis and phylogenetic position of *Cymbomonas tetramitiformis*. It is assumed that the early eukaryotes were prototrophs feeding via phagocytosis (1). This trophic strategy played an important role in the acquisition of a cyanobacterium (2), which was transformed into a primary plastid in the ancestor of Archaeplastida (3). Modern members of this supergroup lost the ability to phagocytose (4) with the exception of some marine prasinophytes including *C. tetramitiformis* (5). On the other hand, heterotrophic eukaryotes lost genes involved in the synthesis of small molecules and became auxotrophic phagotrophs (6). Some of their descendants are still auxotrophic phagotrophs (7), however, others have learned to profit from the prolonged upkeep of their photosynthetic prey (transient endosymbionts) and have become phototrophs (8). These phagocytizing phototrophs could, in the long term, acquire necessary genes via horizontal (including endosymbiotic) gene transfer to tighten the relationships with their endosymbionts following in footsteps of many secondary plastid-containing algae (9). Finally, they could reacquire the lost genes for small compounds, abandon phagotrophy, and become strict phototrophs (i.e. photoautotrophs) (10). The horizontal (including endosymbiotic) gene transfer plays an important role also at all presented stages of the model

It is assumed that the ancestor of Archaeplastida was a unicellular eukaryote with phagotrophic and prototrophic abilities, i.e. it could phagocytize and synthesize small organic molecules (e.g. amino acids, coenzymes, and vitamins) required for its growth, respectively (Fig. 1; Martin and Muller 1998; Martin 2005; Burns et al. 2015). These features helped it to acquire and retain endosymbionts, and in the long term to become fully independent of heterotrophy, i.e. evolve into a strict phototroph (photoautotroph). *C. tetramitiformis* is well-suited to this concept as a transitional form resorting to phagotrophy under phosphate-limited conditions (Paasch et al. 2016). Analyses of its genome have revealed that it has retained genes related to both phagocytic feeding and synthesis of small molecules (Burns et al. 2015). In contrast, heterotrophic phagotrophs lost the latter genes and became auxotrophic, i.e. they cannot synthesize some organic compounds needed for their growth and must obtain them through their diet. According to Burns et al. (2015), these losses prevented, for example, some metazoans, amoebozoans, and ciliates from establishing permanent relationships with their transient photosynthetic endosymbionts (Fig. 1). However, this “transiency” does not have to mean an evolutionary dead end at all. Eukaryotes that have lost genes involved in the synthesis of some small compounds can obtain them by horizontal (and also endosymbiotic) gene transfer (Timmis et al. 2004) according to the “you are what you eat” hypothesis (Doolittle 1998), the “shopping bag” model (Larkum et al. 2007), and the “minor mistargeting” mechanism (Martin 2010).

The uptake of foreign DNA is of great importance for the expansion of metabolic capabilities and as a result can prepare the host to develop a more stable relationship with its transient endosymbiont. There is no doubt that gene transfer was one of the key processes behind the primary plastid establishment (Timmis et al. 2004; Keeling and Palmer 2008; Keeling 2009). Ample evidence for such transfers is found in the highly reduced plastid genomes of Archaeplastida and their mosaic nuclear genomes (Martin et al. 2002; Reyes-Prieto et al. 2006; Deusch et al. 2008; Green 2011). The latter genomes include not only 10–20 % genes of cyanobacterial origin, but also sequences acquired from α-proteobacteria and chlamydia (Martin et al. 2002; Reyes-Prieto et al. 2006; Deusch et al. 2008). The mitochondrial ancestor is responsible for the α-proteobacterial share, however, the gene transfer from chlamydia is much less expected. Recently, it has sparked a very interesting debate and the formulation of the “ménage à trois” hypothesis (Ball et al. 2013). According to Ball et al. (2013) a chlamydia-like symbiont facilitated the acquisition of the cyanobacterial endosymbiont. The chlamydial infection was to provide the enzymes necessary for the host to be able to use the ADP-glucose produced in cyanobacterial photosynthesis, which was inaccessible for its UDP-glucose-dependent glucan synthases (Ball et al. 2013). Moreover, chlamydial effector proteins and transporters could have also helped cyanobacteria to evade the host defence mechanisms and thus proliferate in the cytoplasm (Ball et al. 2016). When all the necessary genes were transferred to the host nuclear genome, a metabolic link that tightened the primary endosymbiosis was forged making the chlamydial co-symbiont redundant (Ball et al. 2013; see also Deschamps 2014 for discussion).

Horizontal (including endosymbiotic) gene transfer from various sources has also facilitated the acquisition of secondary and tertiary plastids (Fig. 1, Larkum et al. 2007), i.e. green and red alga-derived organelles of heterokonts (Bowler et al. 2008; Deschamps and Moreira 2012), chlorarachniophytes (Curtis et al. 2012; Yang et al. 2014), haptophytes (Miller and Delwiche 2015), cryptophytes (Curtis et al. 2012), and dinoflagellates (Patron et al. 2006; Wisecaver et al. 2013; Burki et al. 2014). Interestingly, many members of these lineages still retain the phagotrophic ability like *C. tetramitiformis* does, despite being obligatory phototrophs (Urban 1998; Stoecker et al. 2006; Burkholder et al. 2008; Jeong et al. 2010; Hansen 2011; Unrein et al. 2014; Mitra et al. 2016). The fact that phagotrophic protists often carry many foreign genes suggests that the feeding strategy plays an important role in gene acquisition (Doolittle 1998; Yue et al. 2013; Grant and Katz 2014).

One can argue that a single gene, e.g. involved in the synthesis of small compounds, may not constitute a unit of selection but only the entire set of genes engaged in a metabolic pathway. However, reacquisition of a lost pathway does not have to mean building it from scratch because its ‘remnants’ or homologous genes may function in the host metabolic network. This reasoning is well supported by the phylogenetic mosaicism of metabolic pathways, which, however, can also be explained by gene replacement events, and the general flexibility and robustness of the metabolic network (Curtis et al. 2012; Deschamps and Moreira 2012; Reyes-Prieto and Moustafa 2012; Qiu et al. 2013; Burki et al. 2014; Yang et al. 2014).

Even assuming that all the genes involved in a metabolic pathway are required for plastid establishment, their gain may be as simple as the acquisition of a single gene if the transfer sources are bacteria or unicellular eukaryotes with a simple organization of their genomes. Genes associated with metabolic pathways in prokaryotes are often organised into operons, which highly increases the probability of their simultaneous transfer to a potential host. Such a transfer was described in the case of the parasitic nematode *Heterodera glycines*, which acquired the genes for the synthesis of vitamin B6 from bacteria (Craig et al. 2008). This particular example shows that an entire metabolic pathway can be acquired, even in multicellular organisms.

Although horizontally transferred genes involved in the synthesis of small compounds can transform an auxotroph into a prototroph, it is important to emphasize that prototrophy is not required to maintain a permanent endosymbiont. This trophic ability is only necessary to become a true photoautotroph (strict phototroph). Accordingly, many phototrophic protists with permanent plastids simultaneously feed on eukaryotic microorganisms or bacteria, gain nutrients by other endocytic processes, and perform osmotrophy (Urban 1998; Stoecker et al. 2006; Burkholder et al. 2008; Jeong et al. 2010; Hansen 2011; Unrein et al. 2014; Mitra et al. 2016).

## Acquired phototrophy in the light of the “luggage” hypothesis

The lack of permanent endosymbionts can also be explained by the “luggage” hypothesis proposed by Wouters et al. (2009) on the pages of “Symbiosis” nearly seven years ago. It states that as long as symbionts or plastid donors are present and abundant in the host environment, the most favoured host strategy will be preying on them. Because the prey can be easily acquired from the environment, there is no selective pressure to evolve energy-expensive mechanisms for their permanent upkeep. Therefore some metazoans, amoebozoans, and ciliates may host only transient endosymbionts (Burns et al. 2015). In the case of ciliates, nearly one fourth is estimated to apply this cost-effective strategy. Their kleptoplastids or endosymbionts provide photosynthetic metabolites and oxygen, enabling them to grow faster and find refuge in hypoxic or anoxic waters (Stoecker et al. 2009). On the other hand, the fewer symbionts or plastid donors in the environment, the more likely the host is to hold them as permanent luggage. The hosts that are able to maintain endosymbionts or kleptoplastids for a longer time are favoured, for example, in dynamic environments or when the host finds itself in a new environment (see next section for an example; Wouters et al. 2009).

The only obstacle to establish a permanent endosymbiont or plastid in a unicellular eukaryote is, perhaps, exemplified by foraminiferans. They transmit their endosymbionts vertically during asexual reproduction but the large size of most endosymbionts constrains them from entering gametes. Therefore, the endosymbionts have to be reacquired after gametogamy, resulting in a cyclic nature of the endosymbiotic relationship (Nowack and Melkonian 2010). This kind of interaction is indeed an evolutionary dead end from the endosymbiotic point of view, contrary to auxotrophy suggested by Burns et al. (2015). Given evolutionary time and possibilities, i.e. gene donors, the evolution of endosymbiotic relationships is possible if there is a selective pressure favouring them. The mechanisms driving transient endosymbionts into permanent plastids are described in the “luggage” hypothesis. Although endosymbiosis is sometimes treated as an example of non-Darwinian evolution via the inheritance of acquired characteristics (e.g., the acquisition of new genes), it still comes under the law of natural selection, resulting in the survival (and reproduction) of those best adapted to the environment.

## The case of *Paulinella chromatophora*

Besides *C. tetramitiformis*, another interesting model of endosymbiosis is the thecate amoeba *Paulinella chromatophora*, which harbours two photosynthetic cyanobacteria-derived bodies (called chromatophores), acquired independently of archaeplastidian primary plastids (Bodył et al. 2012; Nowack 2014). The chromatophores are deeply integrated with the host and a substantial number of their genes has been transferred to the host nuclear genome (Nowack et al. 2011). Consequently, a transport system to import these host-encoded proteins has evolved in chromatophores’ membranes (Bodył et al. 2010; Mackiewicz et al. 2012a, b; Nowack and Grossman 2012; Gagat and Mackiewicz 2014). Therefore, the chromatophores should be considered true cell organelles, though they represent an early step of organelle transformation. The case of *Paulinella* fits well into the “luggage” hypothesis as there are marine heterotrophic *Paulinella* species feeding on bacteria (including cyanobacteria) and two photosynthetic *P. chromatophora* strains, FK01 and CCAC 0185, found in brackish and freshwater environments worldwide. That suggests the change of the habitat and consequently the change of the prey supply to be the cause behind the chromatophores’ establishment. Interestingly, another photosynthetic *Paulinella* (*P. longichromatophora*) has recently been discovered in marine sand flats on the western coast of Korea (Kim and Park 2016). This species groups significantly with the two other photosynthetic *Paulinella* on both nuclear and chromatophore rDNA trees indicating a single acquisition of a cyanobacterium by their ancestor. However, the two freshwater *Paulinella* are not monophyletic because *Paulinella* FK01 is closer related to the marine *P. longichromatophora* than to the CCAC 0185 strain. It seems that *P. longichromatophora* returned secondarily to the sea if we assume that the ancestor of the three amoebas lived in freshwater (Kim and Park 2016).

It is noteworthy to mention that contrary to its heterotrophic relatives and also to mixotrophic prasinophytes, *Paulinella* has lost the ability to phagocytose (see Nowack et al. (2011 and references therein). However, taking into account the time that has passed since the primary endosymbiosis (~1500 million years) and *Paulinella* endosymbiosis (~60 – 140 million years), the opposite should be expected (Nowack et al. 2008; Parfrey et al. 2011; Delaye et al. 2016). It is interesting to ponder which selective pressure could be responsible for the maintenance of phagocytosis among prasinophytes. Experimental data indicates that the opportunity to receive additional carbon compounds is not the answer, but combined with the acquisition of other nutrients, such as for example: nitrogen, phosphorus, and iron, it may constitute the selective advantage, especially in oligotrophic (low-nutrient) environments that offer little to sustain life (Flynn et al. 2013; Mitra et al. 2016; Paasch et al. 2016). Such conditions, in general, support mixotrophy as a dominant way of feeding. In accordance with this, another marine phagotrophic prasinophyte *Micromonas* CCMP2099 increases its grazing on bacteria in low light and poor nutrient conditions (McKie-Krisberg and Sanders 2014). In contrast, *P. chromatophora* thrives in small eutrophic (nutrient-rich) ponds. In such environments, phototrophs can easily acquire simple compounds by alternative to phagocytosis endocytic processes and osmotrophy, and therefore do not need to resort to phagotrophy. Nutrient-rich conditions have been shown to drive protist diversification into photoautotrophs and heterotrophs with specialized trophic strategies (Troost et al. 2005).
